# Use of Aligned Microscale Sacrificial Fibers in Creating Biomimetic, Anisotropic Poly(glycerol sebacate) Scaffolds

**DOI:** 10.3390/polym11091492

**Published:** 2019-09-12

**Authors:** Chen-Yu Li, Ming-Hsien Hu, Jin-Jia Hu

**Affiliations:** 1 Department of Biomedical Engineering, National Cheng Kung University, Tainan 701, Taiwan; dorafishing@hotmail.com; 2 Bachelor Program for Design and Materials for Medical Equipment and Devices, Da-Yeh University, Changhua 515, Taiwan; minghsienhu@gmail.com; 3 Orthopedic Department, Showchwan Memorial Hospital, Changhua 500, Taiwan; 4 Department of Mechanical Engineering, National Chiao Tung University, Hsinchu 300, Taiwan

**Keywords:** Poly(glycerol sebacate), electrospinning, sacrificial fibers, anisotropy, contact guidance, small-diameter tissue-engineered vascular grafts

## Abstract

Poly(glycerol sebacate) (PGS) is a biocompatible, biodegradable elastomer that has been shown promise as a scaffolding material for tissue engineering; it is still challenging, however, to produce anisotropic scaffolds by using a thermoset polymer, such as PGS. Previously, we have used aligned sacrificial poly(vinyl alcohol) (PVA) fibers to help produce an anisotropic PGS membrane; a composite membrane, formed by embedding aligned PVA fibers in PGS prepolymer, was subjected to curing and subsequent PVA removal, resulting in aligned grooves and cylindrical pores on the surface of and within the membrane, respectively. PVA, however, appeared to react with PGS during its curing, altering the mechanical characteristics of PGS. In this study, aligned sacrificial fibers made of polylactide (PLA) were used instead. Specifically, PLA was blend-electrospun with polyethylene oxide to increase the sacrificial fiber diameter, which in turn increased the size of the grooves and cylindrical pores. The resultant PGS membrane was shown to be in vitro cyto-compatible and mechanically anisotropic. The membrane’s Young’s modulus was 1–2 MPa, similar to many soft tissues. In particular, the microscale grooves on the membrane surface were found to be capable of directing cell alignment. Finally, based on the same approach, we fabricated a biomimetic, anisotropic, PGS tubular scaffold. The compliance of the tubular scaffold was comparable to native arteries and in the range of 2% to 8% per 100 mmHg, depending on the orientations of the sacrificial fibers. The anisotropic PGS tubular scaffolds can potentially be used in vascular tissue engineering.

## 1. Introduction

Load-bearing tissues, such as blood vessels, heart valves, tendons, and ligaments, generally exhibit structural anisotropy. The anisotropy usually endows these tissues with important, orientation-dependent physiological functions. The anisotropy and the associated functions of these tissues can be compromised by injury, disease, or aging, thereby reducing the quality of life and even causing death. There is thus a great need for restoring functions of these damaged anisotropic tissues.

Tissue engineering, which aims to restore, maintain, or improve tissue function, typically relies on the structural support of a scaffold for initial cell adhesion and subsequent tissue formation. Design criteria for the scaffold include biodegradability, biocompatibility, porosity, and mechanical properties [1]. Creating anatomically correct architecture in tissue-engineered constructs, however, remains challenging [2]. This may be partly attributed to the isotropic nature of conventional scaffolds [3].

A scaffold with biomimetic structural anisotropy can be helpful in regenerating anisotropic, load-bearing tissues, whose mechanical behavior is of importance [4,5]. Several methods have been developed to create anisotropic scaffolds. For instance, mechanically anisotropic, electrospun scaffolds can be made by manipulating fiber alignment during fiber collection [2,6]. Supercritical CO_2_ foaming has been used to create scaffolds containing pores in the foaming direction [7]. Directional freezing has been used to prepare aligned porous scaffolds from synthetic [8,9] or natural [10] polymers.

Biocompatible and biodegradable elastomers have been used to fabricate scaffolds for regenerating soft issues, especially those working dynamically. Poly(glycerol sebacate) (PGS) is a thermoset biodegradable elastomer, synthesized via polycondensation of trifunctional glycerol and bifunctional sebacic acid. Because of its economical synthesis process, biocompatibility, adjustable stiffness and biodegradability, and rubberlike elasticity, PGS has enjoyed great success in many tissue engineering applications [11,12,13,14,15]. In particular, PGS has a modulus ranging from 0.025 to 1.2 MPa, depending on crosslinking intensity [16], which matches well with many biological soft tissues. Typically, for the fabrication of PGS scaffolds, PGS prepolymer (pPGS) that is less crosslinked has to be molded before fully crosslinking. Conventional PGS scaffolds are usually prepared by solvent casting followed by curing and salt leaching—these scaffolds, however, are usually structurally and mechanically isotropic. This may restrict their applications in repairing anisotropic, load-bearing tissues. Because of the thermoset nature of PGS, it remains challenging to make anisotropic PGS scaffolds. Recently, PGS fibrous membranes were fabricated by coaxial electrospinning [17,18,19,20,21,22] and blend electrospinning [23]. These methods, however, are technically demanding or require the optimization of operating conditions.

Recently, we have developed a relatively simple method to fabricate an anisotropic PGS membrane, with the use of aligned sacrificial poly(vinyl alcohol) (PVA) fibers [24]. Briefly, a composite membrane is first formed by embedding aligned PVA fibers in pPGS. The PVA–pPGS composite membrane is then subjected to curing and subsequent PVA removal, thereby resulting in aligned grooves and cylindrical pores on the surface of and within the membrane, respectively, and hence an anisotropic PGS membrane. PVA, however, appears to react with PGS during its curing, altering the mechanical characteristics of PGS. Furthermore, we have failed to demonstrate cell contact guidance using the anisotropic PGS membrane, which may be attributed to the nano-sized grooves/pores. In this study, aligned sacrificial fibers made of polylactide (PLA) were used instead. PLA is biodegradable and commonly used in clinical applications. Its melting point is greater than the curing temperature of PGS, and it does not react with pPGS during curing. Similar to the preparation of aligned PVA fibers, aligned PLA fibers was prepared by electrospinning. Specifically, PLA was blend-electrospun with poly(ethylene oxide) (PEO) to increase the sacrificial fiber diameter, which in turn increased the size of the grooves and cylindrical pores. We postulated that these grooves/pores with increased dimensions might serve as physical cues for cell alignment, which could assist the formation of microstructures in neo-tissues. Particularly, for load-bearing tissue regeneration, this characteristic could help create a more functional tissue-engineered construct [25].

The structural morphology of the sacrificial fibers, PLA-PGS composite membranes, and PGS membrane is illustrated by scanning electron microscopy (SEM). FT-IR was used to verify the removal-specific components during the preparation of the PGS membrane. Uniaxial tensile testing was used to verify the mechanical anisotropy of the PGS membrane. The in vitro cytocompatibility of the PGS membranes was also examined. Finally, based on the same approach, we fabricated a biomimetic, anisotropic, PGS tubular scaffold with a custom-made mold. Its compliance, burst pressure, and suture retention strength were examined.

## 2. Materials and Methods

### 2.1. Materials

Polylactide (Ingeo 3001D) was purchased from NatureWorks (Minnetonka, MN, United States). Poly(ethylene oxide) (M.W. = 100,000) was purchased from Alfa Aesar (Ward Hill, MA, United States). Glycerol and sebacic acid were purchased from Sigma-Aldrich (St. Louis, MO, United States). Dichloromethane (DCM), *N*,*N*-dimethylformamide (DMF), tetrahydrofuran (THF), and chloroform were purchased from Macron (Center Valley, PA, United States). All chemicals were used as received. Dulbecco’s modified Eagle’s medium (DMEM), penicillin/streptomycin, and trypsin-EDTA were purchased from Invitrogen (Carlsbad, CA, United States). Fetal bovine serum (FBS) was purchased from Hyclone (Logan, UT, United States).

### 2.2. Cell Culture

A10 smooth muscle cells (ATCC CRL-1476) were used to evaluate the in vitro cytocompatibility of the membranes. The cells were cultured in DMEM containing 10% FBS, 1% penicillin/streptomycin, and 1% gentamicin. Before reaching the confluence, cells were detached by 0.05% trypsin–EDTA, counted, and resuspended in culture medium for subculture or experiments.

### 2.3. Synthesis of Poly(glycerol sebacate) Prepolymer

PGS prepolymer was synthesized according to the method developed by Wang et al. [26], with modifications. Briefly, equimolar amounts of glycerol and sebacic acid were reacted under nitrogen at 120 °C in a 250 mL three-neck flask, connected to a water trap with agitation for 24 h. The reaction continued at a reduced pressure (40 mTorr) for 6 h. For each preparation, the viscosity of 70% pPGS in THF was verified by a viscometer (DV-E, Brookfield, Middleboro, MA, United States) to be 321.6 ± 16.6 cP to ensure consistency.

### 2.4. Preparation of the Anisotropic Poly(glycerol sebacate) Membrane

The preparation procedure of the anisotropic PGS membrane was similar to that published earlier [24], except that PLA/PEO instead of PVA was used to prepare the sacrificial fibers. First, PEO/PLA blend electrospun membranes were fabricated [27]. Specifically, a mixture of PEO and PLA (3/7 *w*/*w*) was dissolved in a mixture of DCM and DMF (6/4 *v*/*v*), at a total concentration of 20% (*w*/*v*) for electrospinning. Note that the addition of PEO facilitated the electrospinning process, which allowed further increasing the total concentration of polymer solution (from 10% to 20%), thus increasing the diameter of the electrospun fibers (1.57 ± 0.25 μm vs. 2.64 ± 0.45 μm; Supplementary Figure S1) [28]. Note also that in our preliminary study, various ratios of PEO/PLA, including 0/10, 3/7, 5/5, and 7/3 were tested at different total concentrations (10%, 15%, 20%, and 25%), to determine the optimal condition that can consistently produce fibers with the largest diameter. Fibers were collected for 2.5 h to form a membrane (thickness = 227 ± 10 µm; *n* = 5). The rotating speed of the drum was set to be 250 rpm and 1600 rpm for fabricating randomly oriented and aligned PEO/PLA fibrous membranes, respectively; the former was used to make isotropic, control PGS membranes, whereas the latter was used to make anisotropic PGS membranes. As PEO melts at 120 °C, the PEO in the blend fibers was removed by washing the membrane in water. The PLA fibrous membrane was then partially embedded in pPGS by depositing pPGS layer by layer from the bottom up, using 16% pPGS in a mixture solvent (95% ethanol/distilled water: 4/1 (*v*/*v*)) such that the level of pPGS did not exceed the thickness of the fibrous membrane. The resultant PLA-pPGS composite membrane (with PLA fibers on top) was cured in a vacuum oven at 120 °C for 24 h, which rendered the PGS insoluble to chloroform. Finally, the PLA fibers on top of and within the PLA-PGS composite membrane were removed by washing the membrane in chloroform.

PGS solid sheets, as a control, were prepared under the same conditions.

### 2.5. Scanning Electron Microscopy

The morphology of the PLA electrospun membranes, PLA-PGS composite membranes, and PGS membranes was examined by SEM [24]. Briefly, dried samples were mounted, sputter-coated with platinum, and observed in a scanning electron microscope (S-4100, Hitachi, Japan). The cross-section and the longitudinal section of the tubular scaffold were prepared by breaking the scaffold in liquid nitrogen with forceps.

### 2.6. Fourier-Transform Infrared Spectroscopy

The chemical structures of the PEO/PLA blend fibers before and after the removal of PEO, as well as the PLA-PGS composite membrane before and after the removal of PLA, were verified by Fourier transform infrared spectroscopy (FT-IR) [21,22].

### 2.7. Analysis of Distributions of Fiber/Groove Orientation and Fiber Diameter/Groove Width

SEM images of the PLA electrospun membranes, PLA-PGS composite membranes, and PGS membranes were used to assess the distribution of fiber/groove orientation. Briefly, five 256 × 256 images cropped from the SEM images of each group were analyzed by a MATLAB routine, based on a fast Fourier transform algorithm for the distribution of fiber/groove orientation [29]. An alignment index (AI), which is defined as the fraction of fibers lying within 20° of the predominant direction normalized by that of a random distribution (0.22) [30], was used to quantify the distribution of the fiber/groove orientations. Note that values of the AI range from 4.55 for strong alignment (i.e., parallel fibers) to 1.00 for random alignment. On the other hand, three SEM images from each stage of preparation and 30 randomly selected fibers (or grooves) from each image were analyzed for fiber diameter (or groove width) using Image J (National Institutes of Health).

### 2.8. Mechanical Testing of the Poly(glycerol sebacate) Membrane

Uniaxial tensile testing was performed using a custom-made mechanical tester, according to a published protocol [24]. Briefly, dog bone-shaped specimens were punched from the isotropic and anisotropic PGS membranes along and perpendicular to the predominant pore direction, if any, using a miniature ASTM D412-C die. The specimen was stretched cyclically to examine its elasticity. The specimen was stretched to failure if there was no plastic deformation. The Young’s modulus (the slope of a stress–stretch curve between the stretch ratio of 1.2 and 1.4), ultimate strength, and elongation at break were determined from the stress-stretch curve of each specimen (*n* = 5).

### 2.9. Determination of Porosity

The porosity of the membranes was measured by a water displacement method [31]. Briefly, a piece of the membranes was immersed in a 5 mL graduated cylinder filled with a known volume of water, $V_{1}$. When the air in the pores of the membrane was expelled, the increased volume, $V_{2}-V_{1}$, representing the volume of solid material, $V_{solid}$, was measured. The water in the cylinder, $V_{3}$, was measured again after the water-impregnated membrane was removed. The difference between $V_{3}$ and $V_{1}$ was identified as the void volume, $V_{void}$. The porosity was calculated as $\phi =\frac{V_{void}}{V_{solid}+V_{void}}=\frac{V_{1}-V_{3}}{V_{2}-V_{3}}$. Note that a 100 μL micropipette was used with the cylinder for better precision of volume measurement.

### 2.10. Cytocompatibility

A10 cells were cultured in direct contact with the membranes, in order to assess their in vitro cytocompatibility. The Alamar Blue assay was conducted on days 2, 4, and 6 to quantify the metabolic activity of the cells [21]. In essence, living cells reduce Alamar Blue solution, resulting in a color change of the solution. The number of cells was estimated by calculating the percent reduction of Alamar Blue with the following equation:

(1) $$\mathrm{Reduced}\;\mathrm{Alamar}\;\mathrm{Blue}\;(\%)=\frac{A_{562}-(A_{620}\times R_{ο})}{A_{562}}\times 100$$

where $A_{562}$ and $A_{620}$ are the absorbance of test wells at 562 nm and 620 nm, respectively; $R_{ο}$ is the correction factor for filters $=\frac{A_{ο,562}}{A_{ο,620}}$; $A_{ο,562}$ is the absorbance at 562 nm due to oxidized Alamar Blue; and $A_{ο,620}$ is the absorbance at 620 nm, due to the same oxidized Alamar Blue. At least five specimens for each group were analyzed.

### 2.11. Cell Morphology on the Poly(glycerol sebacate) Membranes

The PGS membrane was sterilized by autoclaving at 121 °C (~15 psi) for 20 min. A10 cells were then seeded on top of the membrane at a density of 1 × 10^4^/cm^2^. After one day of culture, the membrane was washed with PBS to remove non-adherent cells, and was subsequently fixed in 2.5% glutaraldehyde for 2 h. The membrane was completely dehydrated through graded alcohol and acetone. Finally, the membrane was cut into small pieces and processed for SEM observation.

### 2.12. Preparation of a Poly(glycerol sebacate) Tubular Scaffold

**Figure 1:**
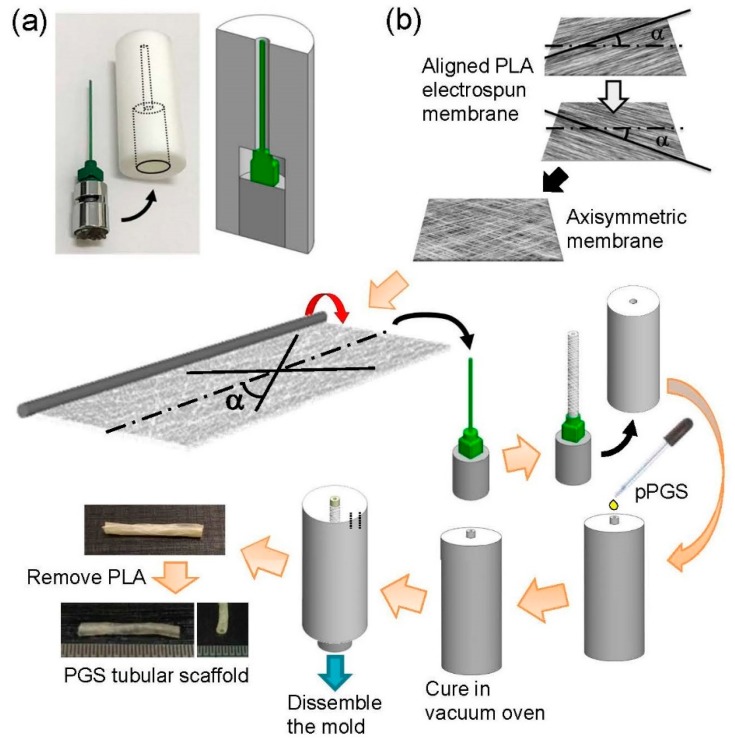
Schematics of the mold assembly (**a**) and the preparation procedure of the poly(glycerol sebacate) (PGS) tubular scaffold (**b**).

Figure 1 shows the preparation procedure of a biomimetic anisotropic PGS tubular scaffold (~3 mm inner diameter). Briefly, rectangular pieces of aligned PLA fibrous membrane were cut in such a way that the angle between the predominant fiber direction and the long axis (i.e., α) was 30° or 90°. Two pieces of such membrane were stacked to form an axisymmetric membrane; the aligned cylindrical pores in each piece aligned symmetrically along the long axis of the rectangle. The axisymmetric membrane was then wrapped around a polytetrafluoroethylene-coated needle (21 G, outer diameter = ~1 mm; 40 mm long), resulting in a sacrificial tubular construct, with a pitch angle α with respect to the axial direction (either 30° or 90°) on the needle. The needle along with the tubular construct was then inserted into a custom-made PTFE tube; essentially, the needle and the PTFE tube form a tubular mold (Figure 1a; for the design of the PTFE tube, the inner diameter of the bottom part should fit the outer diameter of the needle base, so that the coaxial alignment of the needle and the top part of the PTFE tube is ensured). The pPGS was then added to the mold, thereby forming a PLA-pPGS tubular composite in the mold upon the evaporation of the solvent. The whole set was then placed in a vacuum oven (120 °C, 40 mTorr) for 24 h of curing. The cured PLA-PGS tubular composite was then removed from the mold. Note that Vaseline as a mold release was applied onto all surfaces of the mold assembly to facilitate the removal of the tubular construct. Finally, the PLA in the composite was removed by washing in chloroform, resulting in a biomimetic, anisotropic tubular scaffold composed solely of PGS. Tubular scaffolds with different pitch angles (α = 30° and 90°, referred to as A-30 and A-90, respectively) were fabricated.

The mechanical properties of the tubular scaffold were examined using a custom-built mechanical tester [27,32]. Briefly, the tubular scaffold was mounted to a loading frame and immersed in water. After preconditioning, the scaffold was released and re-mounted at its new, unloaded configuration. The scaffold was then subjected to cyclic pressurization from ~10 mmHg to 200 mmHg at three axial stretch ratios (1, 1.05, and 1.1). The compliance of the scaffold was calculated as

(2) $$\mathrm{Compliance}\;(\%\;\mathrm{per}\;100\mathrm{mmHg})=\frac{\left(D_{120}-D_{70}\right)}{D_{70}(120\;\mathrm{mmHg}-70\;\mathrm{mmHg})}\times 10^{4}$$

where *D*_120_ and *D*_70_ are the outer diameter of the scaffold at 120 mmHg and 70 mmHg, respectively.

The burst pressure and suture retention strength of the tubular scaffold were examined using the same mechanical tester, following the protocol described previously [32].

### 2.13. Statistical Analysis

Data are presented as mean ± standard deviation. One-way ANOVA in conjunction with Tukey post hoc procedure was performed to examine the difference in the mechanical properties of the PGS membranes, due to the orientation of cylindrical pores. Student’s *t*-test was used to compare the difference in the compliance, burst pressure, and suture retention strength of the PGS tubular scaffolds, due to the pitch angle. The level of significance was set at 0.05. A * denotes *p* < 0.05, while ** denotes *p* < 0.001.

## 3. Results and Discussion

**Figure 2:**
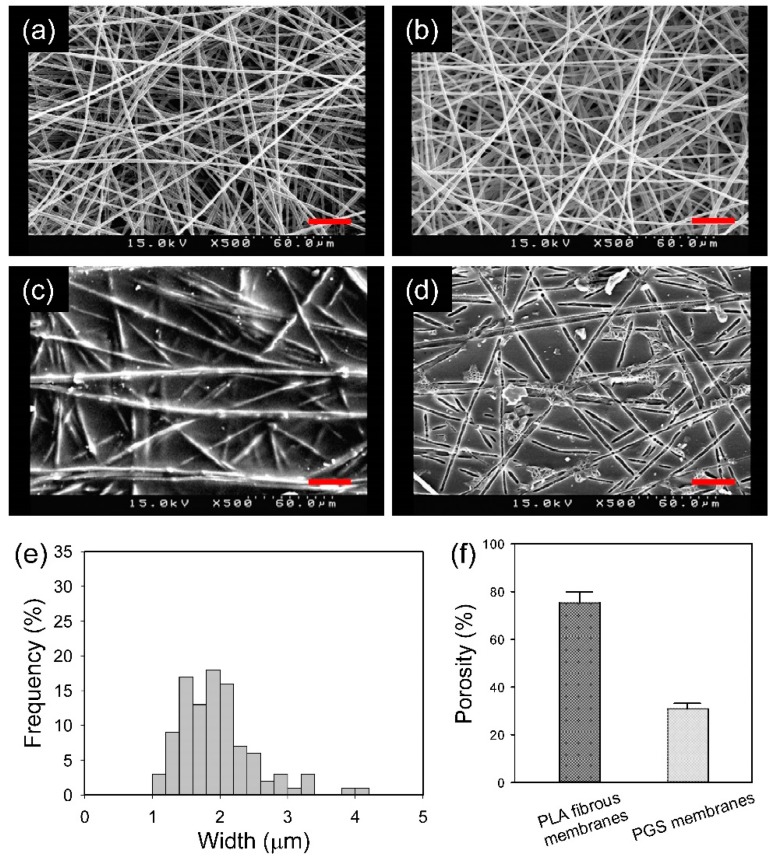
Scanning electron microscopy (SEM) images of the as-spun poly(ethylene oxide) (PEO)/ polylactide (PLA) fibrous membrane (**a**), the PEO/PLA fibrous membrane after removing the PEO (**b**), the PLA-PGS composite membrane (**c**), and the PGS membrane (**d**). The distribution of groove width on the PGS membrane (**e**). The porosity of the PLA fibrous membrane and PGS membrane (**f**). Scale bar = 30 μm.

The SEM images of the PGS membranes at different stages of the preparation are shown in Figure 2. PEO/PLA blend fibers that were collected on the drum rotating at 250 rpm were randomly oriented. The morphology of the blend fibers was not affected by the water washing. There were still PLA fibers over the PLA-PGS composite membrane; that is, the PLA fibers were not fully embedded in PGS. This is a necessary condition for creating grooves on the PGS membrane surface. Washing the composite membrane with chloroform revealed grooves on the surface. The groove width was consistent with the PLA fiber diameter (Figure 2e). The distribution of groove width was slightly broader than that of the diameter of the PLA fibers, because the grooves were in fact the longitudinal sections of fibers cut at different levels of each fiber. The groove width was in the range of 1–3 μm, significantly greater than that of our previous (isotropic or anisotropic) PGS membrane, which was prepared with the use of sacrificial PVA fibers (~200 nm) [24].

The porosity of the PGS membranes was about 30% (Figure 2f). Supposedly, the void volume of the PLA fibrous membrane was completely filled by PGS; that is, the addition of porosity of the PLA fibrous membrane and that of the PGS membrane should be equal to one. The mismatch may be due to the preparation process, in which the PLA fibrous membrane was compressed (i.e., its porosity was reduced), resulting in a PGS membrane with a porosity greater than expected.

**Figure 3:**
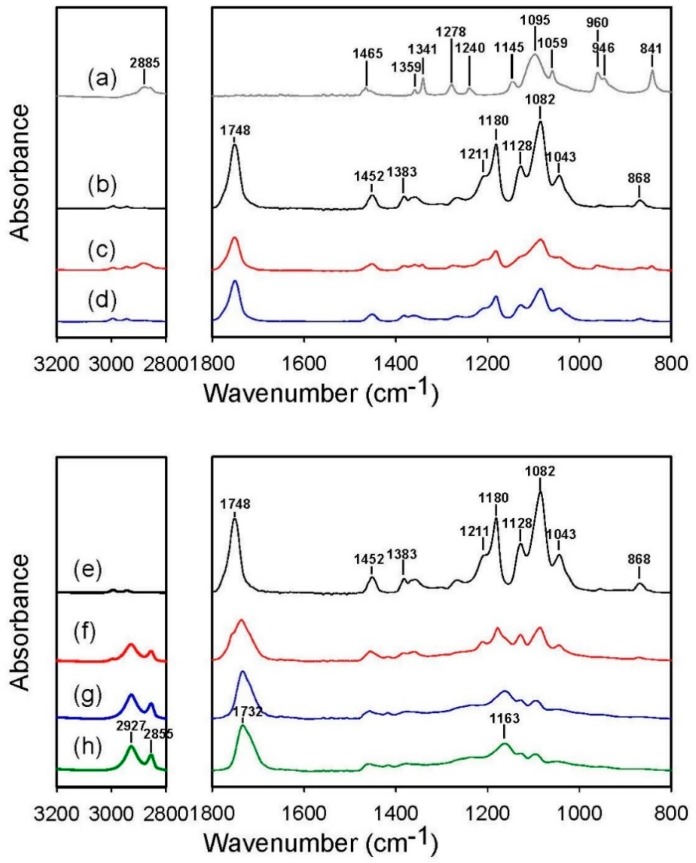
IR spectra of the electrospun PEO fibers (**a**), the electrospun PLA fibers (**b** and **e**), the as-spun PEO/PLA blend fibers (**c**), the blend fibers upon the removal of PEO (**d**), the PLA-PGS composite membrane (**f**), the composite membrane upon the removal of PLA (**g**), and the PGS solid sheet (**h**). Note that the all PGS samples were cured under 120 °C for 48 h.

FT-IR spectroscopy was used to examine the effectiveness of the removal of PEO or PLA, as it allows the identification of functional groups. When comparing the infrared spectra of the as-spun PEO/PLA blend fibrous membrane and the membrane being washed by water, we found that the washing led to the disappearance of absorbance peaks specific to PEO (1145, 1095, and 1059 cm^−1^ (C–O–C stretching); 2885 cm^−1^ (CH_2_ stretching); 1341 cm^−1^ (CH_2_ wagging); 1278 cm^−1^ (CH_2_ twisting); and 841 cm^−1^ (CH_2_ rocking)) and exposed the absorbance peak at 868 cm^−1^ that is specific to PLA (Figure 3, top panel). This indicates that PEO was removed after washing. Similarly, when comparing the infrared spectra of the PLA-PGS composite membrane and the membrane being washed by chloroform, we found that the washing reduced the absorbance specific to PLA (1748 cm^−1^ (C = O stretching specific to PLA, in contrast to 1732 cm^−1^ that belongs to cured PGS); 1211, 1180, and 1082 cm^−1^ (C–O–C stretching); 1128 cm^−1^ (CH_3_ deformation); 1043 cm^−1^ (C–CH_3_ stretching); 868 cm^−1^ (C–COO stretching); and 758 cm^−1^ (δC = O in-plane bending)) (Figure 3, bottom panel). This indicates that PLA was removed after washing.

Anisotropic scaffolds are usually made in the form of aligned fibrous scaffolds or scaffolds containing directional pores. Although pure PGS fibers have been made successfully by coaxial electrospinning [21,22], the fabrication process is complicated and time-consuming. PGS scaffolds are usually made by solvent casting and salt leaching. Because of the isotropic nature of the fused salt template, PGS scaffolds are structurally and mechanically isotropic. This isotropy may restrict their tissue engineering applications, as many tissues in the body, particularly load-bearing tissues, have anisotropic structures and mechanical behavior.

**Figure 4:**
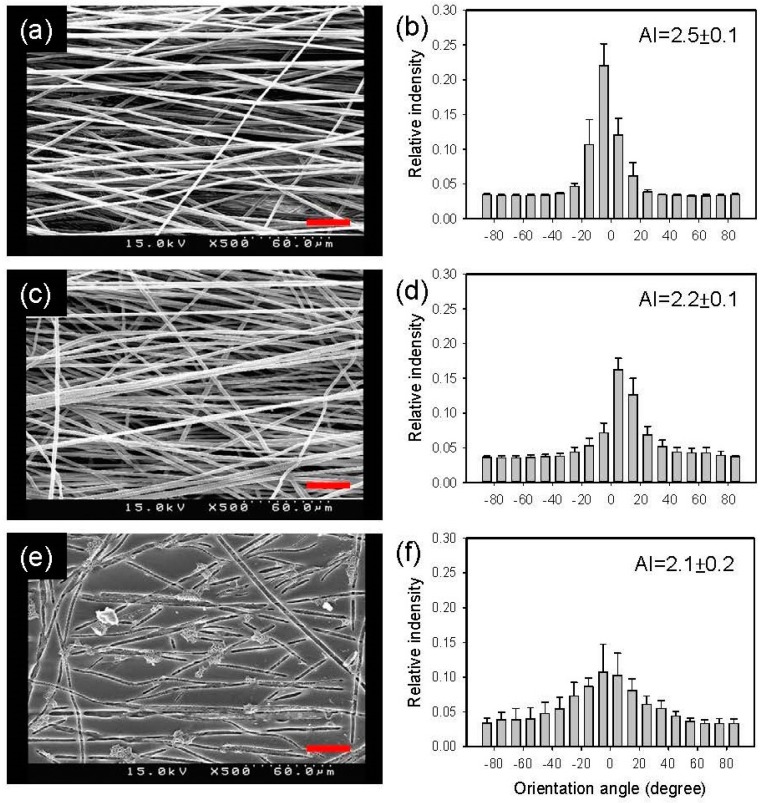
SEM images of the aligned, as-spun PEO/PLA fibrous membrane (**a**), the aligned PEO/PLA fibrous membrane after removing the PEO (**c**), and the aligned PGS membrane (**e**). (**b**,**d**,**f**) The corresponding distributions of fiber/groove orientation. Scale bar = 30 μm.

Aligned sacrificial fibrous membranes were prepared and used to fabricate the anisotropic PGS membrane. The SEM images of the anisotropic PGS membranes at different stages of the preparation are shown in Figure 4. The alignment of PEO/PLA blend fibers was clearly observed when fibers were collected at 1600 rpm. The alignment became less strong after washing with water. Nevertheless, the alignment of grooves on the PGS membrane surface persisted after curing, and the PLA removal and was consistent with the aligned sacrificial fibers. Note that the PLA-PGS composite membrane was not analyzed for alignment index because of ill-defined fiber morphology at this stage.

**Figure 5:**
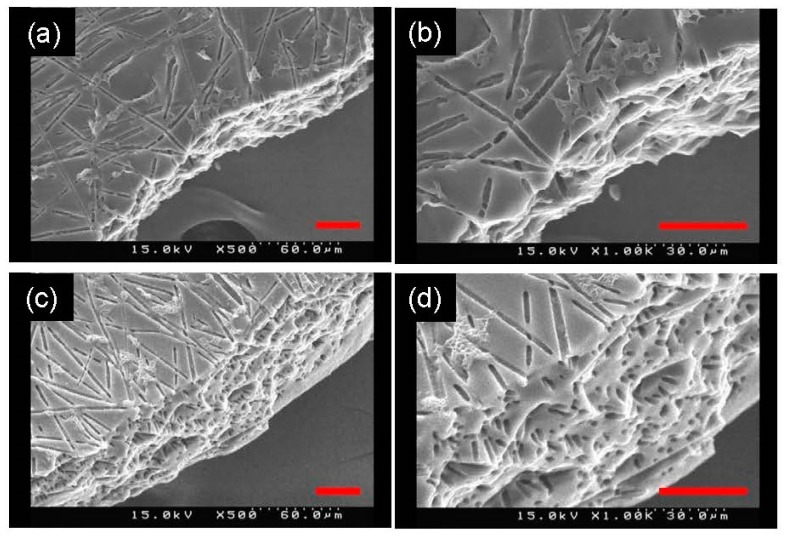
SEM images of the fracture surface of the isotropic PGS membrane (**a**,**b**) and the anisotropic PGS membrane (**c**,**d**). (**b**) and (**d**) show part of (**a**) and (**c**) in higher magnification, respectively. Scale bar = 30 μm.

To verify the removal of PLA fibers inside the PGS membrane, a fracture surface was prepared by breaking the membrane in liquid nitrogen and observed by SEM. There were numerous holes on the fracture surface of the PGS membrane, particularly on the anisotropic PGS membrane, as shown in Figure 5. The presence of holes on the fracture surface indicates the successful removal of PLA fibers inside the membrane; that is, PLA fibers in the PGS membrane were replaced by cylindrical pores.

**Figure 6:**
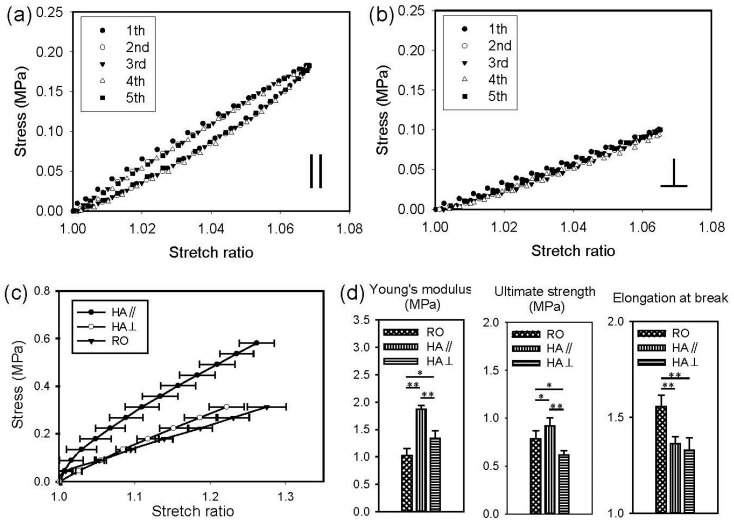
Representative cyclic stress-stretch curves of the anisotropic PGS membranes tested in parallel (**a**) and perpendicular (**b**) to the predominant pore direction. (**c**) Mean stress-stretch curves of the isotropic and the anisotropic PGS membranes in uniaxial tensile testing. (**d**) Comparisons of the Young’s modulus, ultimate strength, and elongation at break of the PGS membranes.

Uniaxial tensile properties of the anisotropic PGS membrane were determined both in parallel and perpendicular to the predominant pore direction. As shown in Figure 6, when the anisotropic PGS membrane was stretched in either direction up to a stretch ratio of 1.1, it returned to its original shape without plastic deformation upon unloading. This again suggests that PLA, which is much stiffer, was completely removed. The five cycles of loading and unloading curves were nearly the same. However, he loading and unloading curves did not coincide with each other, particularly when the membrane was tested in the predominant pore direction, illustrating pseudo-elastic behavior.

Figure 6 also shows the results of mechanical testing when the membranes were stretched to failure in either direction. The stress-stretch behavior of the anisotropic PGS membrane (HA, or highly aligned), regardless of the stretching direction, was similar to that of a typical elastomer. The Young’s modulus and ultimate strength of the anisotropic PGS membrane in the predominant pore direction (||) were greater than those perpendicular to the pore direction (⊥), indicating anisotropic mechanical behavior. There was no significant difference in the elongation at break between the two stretching directions, however. The results of statistical analysis also showed that the Young’s modulus, ultimate strength, and elongation at break of the isotropic PGS membrane (RO, or randomly oriented) are significantly different from those of the anisotropic PGS membrane tested in either direction. The Young’s modulus of the isotropic and anisotropic PGS membranes was in the range of 1–2 MPa for each, similar to that of native blood vessels (0.6–3.5 MPa) [33]. Note that the Young’s modulus, ultimate strength, and elongation at break of our previous (isotropic or anisotropic) PGS membrane were greater than those of the present (isotropic or anisotropic) PGS membrane [24]. Also, the mechanical contribution of the pore structure was different in the two studies. For example, the *aligned* pore structure had no effects on the ultimate strength of the previous PGS membrane [24]; the potential reaction of PVA and pPGS during curing could mechanically strengthen the cylindrical pore structure because of the stiffness of dry PVA.

**Figure 7:**
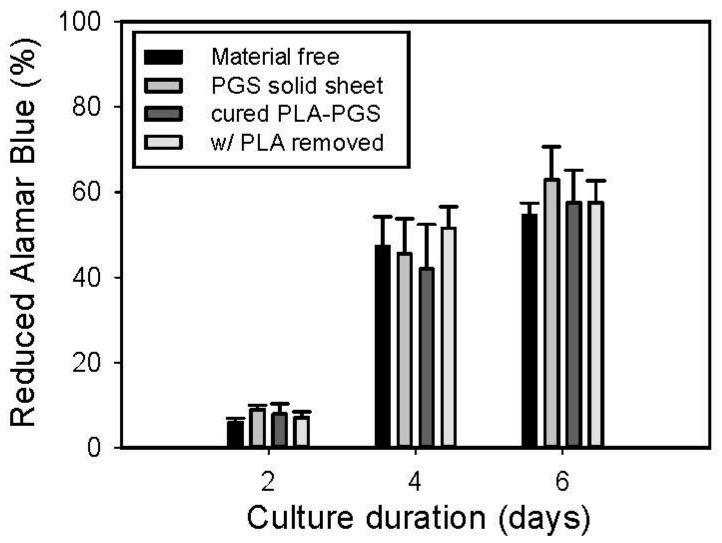
In vitro cytocompatibility of the cured PLA-PGS composite membrane and that of the anisotropic PGS membrane. A10 cells cultured in the medium (i.e., material-free) and with the cured PGS solid sheet were the two negative controls.

The in vitro cytocompatibility of the anisotropic PGS membrane monitored by Alamar Blue assay is shown in Figure 7. The number of A10 cells grown in contact with the cured PLA-PGS composite membrane, anisotropic PGS membrane, and cured PGS solid sheet, as well as those simply grown without adding any materials, was not significantly different at 2, 4, and 6 days.

**Figure 8:**
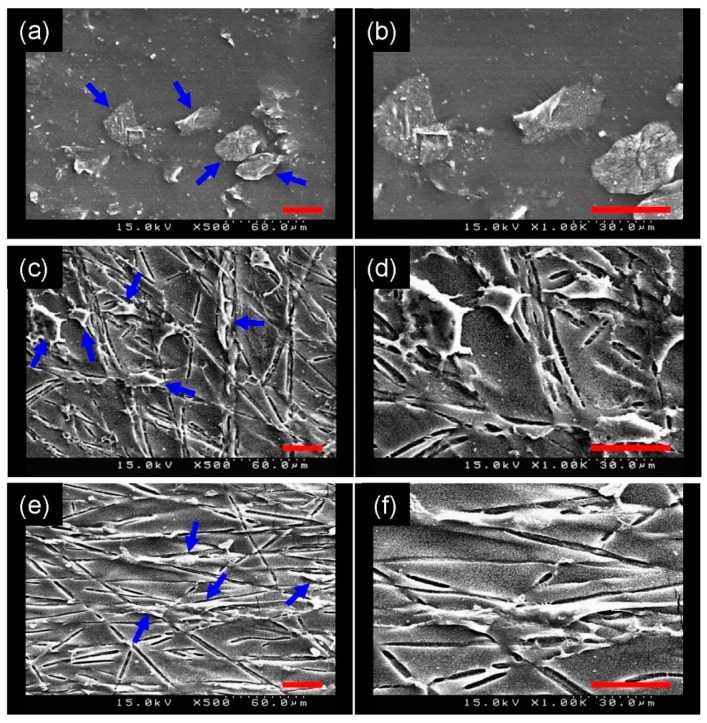
Representative SEM images of A10 cells (blue arrows) seeded on the PGS solid sheet (**a**,**b**), on the isotropic PGS membrane (**c**,**d**) and on the anisotropic PGS membrane (**e**,**f**). Panels (**b**), (**d**), and (**f**) show part of panels (**a**), (**c**), and (**e**) in higher magnification, respectively. Scale bar = 30 μm.

Because of the microscopically uneven flatness of the membrane surface, SEM instead of fluorescence microscopy was used to demonstrate the capacity for cell contact guidance of the anisotropic PGS membrane. Figure 8 illustrates that the A10 cells exhibited spindle-shaped morphology and aligned to the surrounding groove when seeded on the anisotropic PGS membrane, whereas the cells seeded on the isotropic PGS membrane displayed a stellate-like morphology, indicating the ability of the anisotropic PGS membrane to align cells.

Topographical features of scaffolds are beneficial, in that cell alignment and hence tissue microstructure may be manipulated by the mechanism of contact guidance [27,34,35]. Sarkar et al. found that grooves with widths ranging between 10 and 100 μm significantly enhance cell aspect ratio and alignment [36]. Similarly, Bettinger et al. created topographically micropatterned PGS substrates for contact guidance [37]. By decreasing the dimensions of topographical features, Loesberg et al. found that cells are still aligned to grooves down to cut-off values of 100 nm in width and 75 nm in depth [38]. The width of grooves on the PGS membranes herein, determined by the diameter of the PLA fibers, was about 2 µm, which is indeed in the range of proper contact guidance.

**Figure 9:**
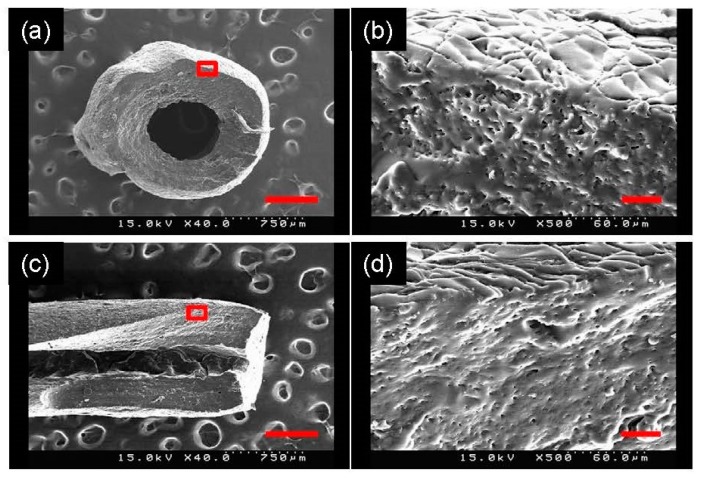
SEM images of the cross section (**a**) and the longitudinal section of the PGS tubular scaffold. Panels (**b**) and (**d**) are the magnified view of the red rectangular region in panels (**a**) and (**c**), respectively. Scale bar = 500 μm in (**a**) and (**c**); scale bar = 30 μm in (**b**) and (**d**).

PGS has been used in fabricating scaffolds for vascular tissue engineering [12,39,40]. Most of these scaffolds have an isotropic media layer; for example, seamless tubular scaffolds were fabricated by solvent casting and salt leaching [39]. The SEM images of the cross-section and the longitudinal section of the PGS tubular scaffold is shown in Figure 9. The image at lower magnitude illustrated a uniform circular lumen with a consistent wall thickness (~0.4 mm). The image at higher magnitude demonstrated the presence of grooves and pores on the external surface and fracture section of the tubular scaffold, respectively.

**Figure 10:**
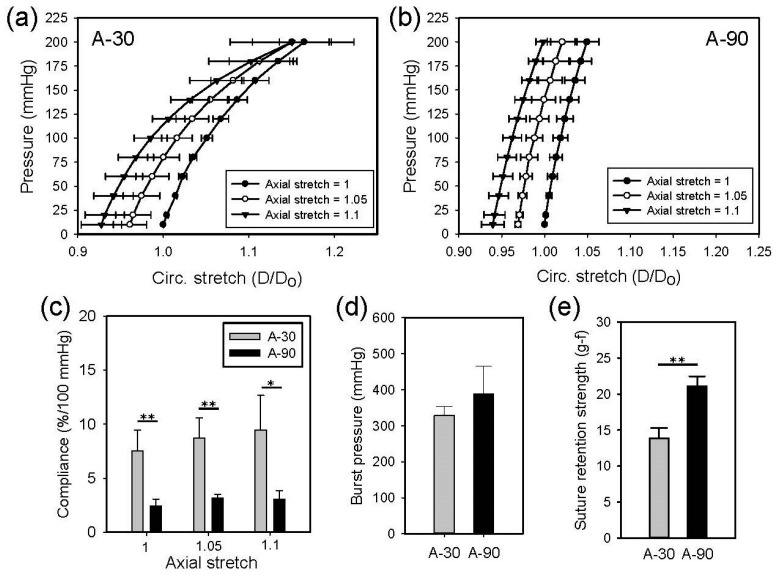
Mean pressure-diameter curves of A-30 (**a**) and A-90 (**b**) at different axial stretches, as well as the comparison of their compliance (**c**), burst pressure (**d**), and suture retention strength (gram-force) (**e**).

Figure 10 shows the results of mechanical testing of the A-30 and A90 tubular scaffolds. The slope of the pressure-diameter curves of the A-30 scaffolds tested at three axial stretches were smaller than those of the A-90 scaffolds tested similarly; the A-30 scaffolds were significantly more compliant than the A-90 scaffolds at each axial stretch. Specifically, the compliance of the A-30 scaffolds was in the range of 5–10% per 100 mmHg, which was similar to native arteries [41]. Axial stretching appeared to increase the compliance of the A-30 scaffolds slightly, but not the A-90 scaffolds. The burst pressure of the A-30 and the A-90 scaffolds were not significantly different. The suture retention strength of the A-90 scaffolds was significantly greater than that of the A-30 scaffolds.

In our previous study, tubular scaffolds were prepared by wrapping a stack of axisymmetric anisotropic PGS membranes around a mandrel [24]. A thin layer of electrospun fibers had to be deposited on the external surface of the scaffold to avoid unwrapping the membranes. This approach requires a significant amount of culturing time for the seeded cells to integrate the layered structure, hindering the possibility of acellular implantation. The compliance of the previous A-30 tubular scaffolds is in the range of 5–8% per 100 mmHg, which is comparable to that of the presented A-30 scaffolds. It has been proposed that residual PVA in the PGS fibers that were obtained from PGS-PVA core-shell fibers leads to different mechanical behaviors of the dry and wet PGS fibers [17]. Note that in the previous and present studies, the PGS membranes were mechanically tested in dry conditions, whereas the PGS tubular scaffolds were tested when they were immersed in water. This may explain the controversy when comparisons of the anisotropic PGS membrane and the PGS tubular scaffold between the two studies have been made.

There are a few limitations in this study. The porosity and pore size of the PGS scaffold was far smaller than that of conventional scaffolds. As wider grooves are desired to facilitate contact guidance of cells, we managed to obtain thicker sacrificial fibers, which inevitably increased the porosity of the sacrificial fibrous membrane and thus decreased the porosity of the PGS membrane. Note that the porosity of the PGS membrane was determined by the ratio of the sacrificial fibrous membrane to the volume of the composite membrane. The porosity of the PGS scaffold may be increased by, for example, mechanically compressing the sacrificial fibrous membrane. Note that further increasing the porosity of the PGS scaffold may decrease the mechanical strength of the scaffold. Secondly, the ideal material for the sacrificial fibers should sustain the curing temperature without melting or degradation, not react with pPGS, and be easily removed. In this study, PLA was selected because of its inertness and stability at the curing temperature. The use of organic solvents like chloroform, however, is required for its removal. Note that the selective removal of PLA is challenging, because both PLA and PGS are polyesters. Finally, while the burst pressure of the PGS scaffold was greater than systemic arterial pressure, the suture retention strength of the PGS scaffold was not sufficient for surgery. This issue may be resolved by depositing a layer of fibrous polymer external to the scaffold [12,40]. There is a need to clarify the relationship between an anisotropic scaffold and the structure and mechanical properties of the derived tissue. In a future study, we will implant the acellular PGS tubular scaffold as a vascular bypass graft, and examine the anisotropy of the scaffold on the remodeling outcome of the implant in vivo.

## 4. Conclusions

In this study, we used PLA, which does not react with PGS, to fabricate aligned sacrificial fibers for the preparation of the anisotropic PGS membrane. Specifically, PLA was blend-electrospun with PEO to increase the sacrificial fiber diameter, which in turn increased the size of the grooves and cylindrical pores on the surface of and within the membrane, respectively; the spaces that were originally occupied by PLA fibers became grooves and cylindrical pores upon their removal. The PGS membrane was shown to be cytocompatible in vitro and mechanically anisotropic. The Young’s modulus of the PGS membrane was in the range of 1–2 MPa, which was similar to many soft tissues. In particular, the microscale grooves on the membrane surface were found to be capable of directing cell alignment. Finally, based on the same approach we fabricated a biomimetic anisotropic PGS tubular scaffold, the compliance of which was comparable to native arteries and in the range of 2% to 8% per 100 mmHg, depending on the orientations of the sacrificial fibers. The anisotropic PGS tubular scaffolds have the potential to be used in vascular tissue engineering.

## Supplementary Materials

The following are available online at https://www.mdpi.com/2073-4360/11/9/1492/s1, Figure S1: SEM images of as spun PLA fibers (a), PEO/PLA blend fibers (c), and blend fibers with the PLA removal (e), and the corresponding distributions of fiber diameter (b,d,f). Note that 10% (w/v) PLA solution was used for preparing the as spun PLA fibers.
