# Supplementary material for: Use of Aligned Microscale Sacrificial Fibers in Creating Biomimetic, Anisotropic Poly(glycerol sebacate) Scaffolds
Source: Polymers (Basel). 2019 Sep 12;11(9):1492. doi: 10.3390/polym11091492 (PMC6780144; doi:10.3390/polym11091492)
Supplement: Supplementary file 1 [file polymers-11-01492-s001.pdf]

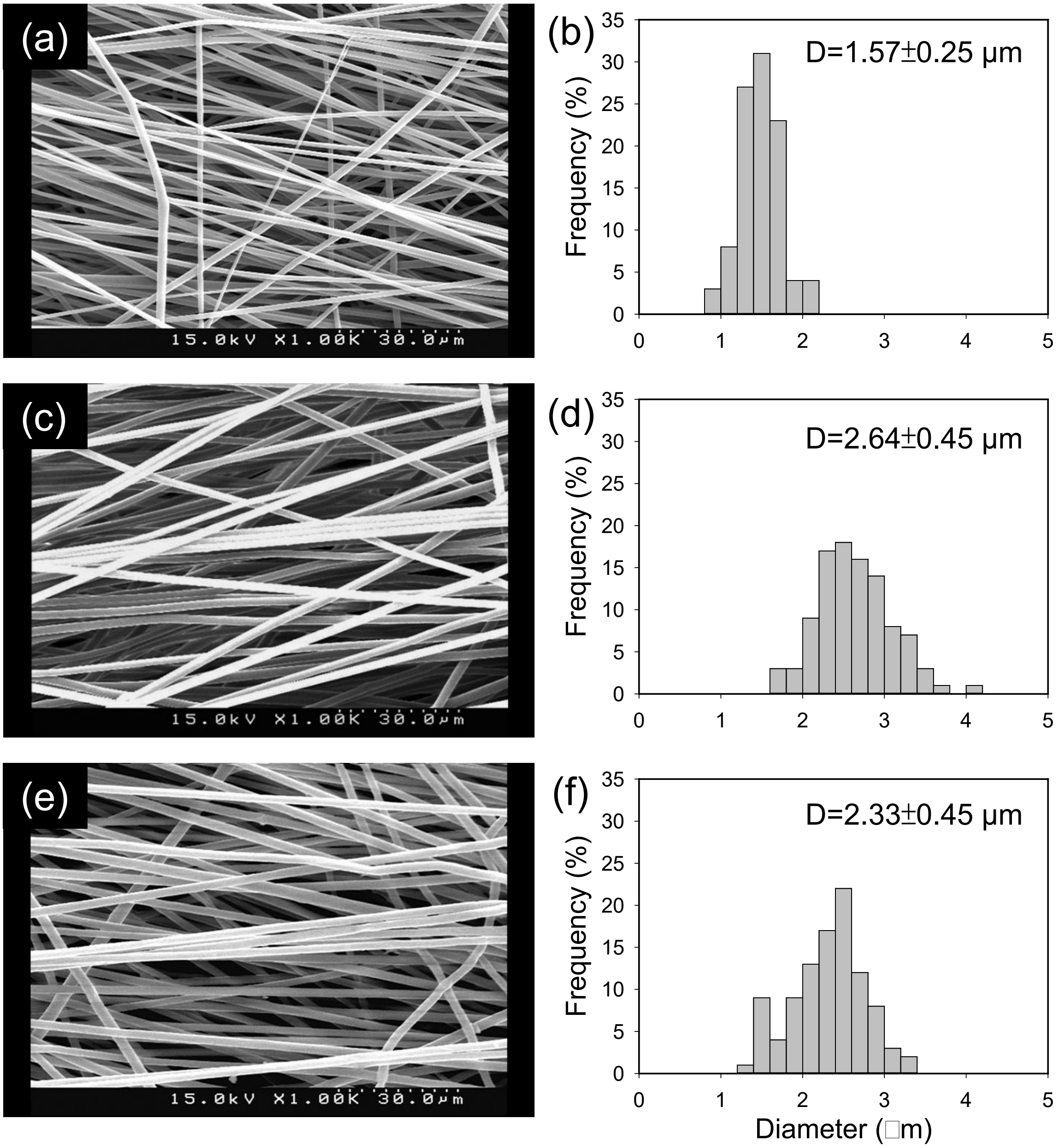


Supplementary Figure 1. SEM images of as spun PLA fibers (a), PEO/PLA blend fibers (c), and blend fibers with the PLA removal (e), and the corresponding distributions of fiber diameter (b, d, f). Note that 10% (w/v) PLA solution was used for preparing the as spun PLA fibers.
